# Valorization of Water Treatment Sludge for Applications in the Construction Industry: A Review

**DOI:** 10.3390/ma17081824

**Published:** 2024-04-16

**Authors:** Ana Paula Mattoso, Sandra Cunha, José Aguiar, António Duarte, Helena Lemos

**Affiliations:** 1 CTAC—Centre for Territory, Environment and Construction, University of Minho, Campus of Azurém, 4800-058 Guimarães, Portugal; id10627@alunos.uminho.pt (A.P.M.); sandracunha@civil.uminho.pt (S.C.); aduarte@civil.uminho.pt (A.D.); 2 Águas do Norte, S.A., Dom Pedro de Castro, 5000-669 Vila Real, Portugal; helena.lemos@adp.pt

**Keywords:** water treatment sludge, waste recovery, circular economy, sustainable constructions

## Abstract

To address the growing global water demand, it is imperative to implement advanced treatment systems and sustainable alternatives for managing the large amount of waste generated during the water purification process, known as water treatment sludge (WTS). Worldwide, researchers and companies are exploring alternatives and methods for the valorization of WTS as a raw material in other processes. It is urgent that all productive sectors, which contribute significantly to greenhouse gas emissions, adopt this management principle to ensure more sustainable production, contributing to the global goal of climate neutrality. Notably, in civil construction, incorporating WTS as a supplementary cementitious material (SCM) shows great promise, considering that the industrial waste currently used for this purpose is increasingly restricted. The use of WTS as a raw material in the cement industry not only contributes to the reduction of the carbon footprint, but also reduces the high waste load still disposed of in landfills. The emerging applications for WTP sludge are reviewed, with emphasis on its valorization in the civil construction as an SCM. The main characteristics of this waste and their impacts on the environment are also addressed.

## 1. Introduction

### 1.1. General Considerations

As the world’s population grows, water consumption is expected to double by 2050 [1] and to meet the new increasing demand, water treatment plants (WTPs) will need to increase their production of drinking and industrial water, which will mean generating a greater amount of waste from treatment process, known as water treatment sludge (WTS). On a global scale, it is estimated that more than 10,000 tons of sludge are generated per day [2]. Management practices for this waste generally differ between countries, depending on local regulations, but the most common destination is landfills, which has a negative impact on the environment. For this reason, the search for sustainable alternatives for the treatment and reuse of WTS has become an object of study for researchers all over the world [3] and one of the options is to use it as a raw material in other production processes, applying the principle of the circular economy. This practice will help to minimize the problems of natural resource scarcity faced nowadays.

On the other hand, to guarantee sustainable production, decarbonizing production chains has become a global challenge. One of the strategies adopted by the European Union (EU) to overcome this challenge and achieve the goal of zero net greenhouse gas (GHG) emissions by 2050 is to encourage more efficient use of natural resources, mobilizing industry towards a circular and clean economy [4]. As a large part of greenhouse gas emissions come from the extraction and processing of natural resources and considering that the amount of waste generated in the world is tending toward increasing significantly, expanding the implementation of a circular economy among the various productive sectors is an essential measure for achieving the global goal of climate neutrality. It is in this context that the waste generated in water treatment plants appears to be a promising alternative to be valorized as a raw material in various sectors, since WTS has added value; it comes from an essential activity and is therefore always available; and it can be recycled. In this respect, the construction sector is one of the biggest consumers of natural resources, with a high environmental impact, mainly associated with the production of cement [5,6,7]. Some alternatives to reduce the impact of production have already been proposed, such as alternative raw materials, alternative fuels, energy recovery and new clinker compositions and geopolymer binders [8]. However, these options often become unfeasible due to technical, operational and regulatory issues [9]. The use in the manufacture of clinker, for example, comes up against the problem of material availability, since these minerals are industrial by-products and are increasingly restricted to supply cement demand [10,11,12,13]. In the case of fly ash, the industry that supplies the waste is gradually being phased out. Around 40% of coal-fired power stations in the US have been decommissioned in the last five years; the UK plans to decommission all its coal power stations by 2025 [14], while in the Netherlands, coal-fired power stations will be decommissioned by 2030 [15]. Another option pointed out by researchers in the field is the use of more efficient cements, such as low-heat portland (LHP) cement, which has a wide range of applications and is considered to have satisfactory performance [16].

In addition, construction materials use large quantities of cement, the production of which not only requires the extraction of non-renewable minerals, but is also responsible for high emissions of carbon dioxide (CO_2_). In this way, introducing WTS into the construction industry chain to partially replace cement, as a supplementary cementitious material (SCM), will reduce the use of this binder and positively impact the global goal of zero net greenhouse gas emissions. Research carried out by a working group supported by the United Nations Environment Program Sustainable Building and Climate Initiative (UNEP-SBCI) also identified the use of MCS to partially replace clinker or as a partial substitute for cement in construction materials as the most favorable carbon reduction alternative for the industry [10].

Therefore, considering the scenario of the global plan for sustainable development defined in the 2030 Agenda of the United Nations (UN), the subject of this research review is inserted in the following Sustainable Development Goals (SDGs):

SDG 6: Ensure sustainable drinking water for all;

SDG 9: Sustainable industries and infrastructures;

SDG 11: Building sustainable and resilient cities;

SDG 12: Efficient use of natural resources and environmentally sound waste management.

The aim of this work is to present an overview of the alternatives for recovering WTP sludge in the context of the circular economy, with an emphasis on applications for this waste in the construction industry. The main characteristics of WTS and its environmental impacts will also be addressed in this research.

### 1.2. Scope of the Review Paper

The methodology employed in this review prioritized papers published in the last 15 years to ensure the review remains up to date and presents a comprehensive perspective on the research conducted in this area.

The review is divided into four sections. Section 1 presents a brief description of the potential for recovering the waste generated in water treatment plants as a raw material for other industries, with special attention to the construction industry, highlighting the challenges faced by the sector today and the context in which the subject of this review is inserted. Section 2 presents the main physicochemical characteristics of WTS and the negative environmental impacts that its improper disposal can cause. In the same section, some properties of cement and other pozzolanic materials are also presented, for comparison purposes and to prove the viability of using WTS. Section 3 portrays the applications for WTS in different sectors, with a more detailed approach leading to its valorization in the construction industry, presenting different subsections that include cement manufacture, mortar and concrete production, the manufacture of tiles and ceramic products and the application of sludge in geotechnical works. This highlights the huge potential for using this waste as a raw material for industry. Finally, Section 4 highlights the conclusions of this review and Section 5 includes some recommendations regarding new approaches for future studies in the area.

## 2. Main Characteristics of Sludge Generated in Water Treatment Plants

Ensuring safe drinking water for all is among the Sustainable Development Goals put forward by the UN as one of the targets needed to ensure the health and well-being of humanity. Therefore, to achieve the goal of universalization of this service by 2030, it will be necessary to implement more producer systems and from this perspective, sanitation companies and governments will also need to implement sustainable alternatives for the treatment and disposal of the large amount of sludge that will be generated in the process. WTS is waste generated during the process of treating drinking water, consisting of water and suspended solids originally contained in the source of supply, plus products resulting from the reagents applied in the treatment. Some of the chemical products commonly used in WTPs as coagulants/flocculants include aluminum sulphate, ferric chloride, chlorinated ferrous sulphate, ferric sulphate, aluminum hydroxy-chloride, synthetic polymers (cationic, anionic and non-ionic) and natural polymers (cassava and potato starch) [2,17].

The qualitative and quantitative characteristics of this waste depend on the quantity and quality of the water that feeds the plant, the treatment technology used and the chemicals and dosages applied in the process. [18]. Thus, colloidal particles and suspended materials such as silt, clays, humic substances and metals, among other impurities originally present in the raw water, and chemicals in the form of aluminum or iron hydroxide, using salts of these metals as coagulants, will make up the solid fraction of these sludges. Silica (SiO_2_), alumina (Al_2_O_3_) and hematite (Fe_2_O_3_) generally account for a significant proportion of these solids [19]. However, other oxides such as calcium oxide (CaO), magnesium oxide (MgO), sodium oxide (Na_2_O), potassium oxide (K_2_O), phosphorus pentoxide (P_2_O_5_) and sulphur trioxide (SO_3_), as well as traces of other metals, chlorides (Cl^−^), sulphates (SO_4_^2−^) and other organic and inorganic compounds, removed from the raw water or added as impurities contained in the chemical products, will also be present in the composition of the sludge. The moisture content of this waste is usually over 80% by weight and the organic matter content is around 25% [20].

When aluminum salt coagulants are used, the waste generated is known as “alum sludge”, but if the coagulant used is based on iron salts, the waste obtained is called “ferric sludge” [2]. Alum sludge is more common, because aluminum sulphate and aluminum chloride are the most used coagulants in water treatment [21,22] and the aluminum content in these sludges generally represents 16% by weight of their chemical composition [23]. Whatever its origin, the characterization of the waste is essential to define the best way to handle and use it, given that its composition varies greatly [3]. Table 1 shows the elemental chemical composition (main oxides) of WTS generated in some countries. These chemical characteristics affect the options for reuse and final disposal more than the ability to handle and dewater.

WTS has high humidity levels and to facilitate its handling, it is extremely necessary to dewater it. Both alum sludge and ferric sludge are considered difficult to dewater when they have specific resistance to filtration values between 5 × 10^12^ m/kg and 50 × 10^12^ m/kg [24] and to facilitate the dehydration of this waste, polyelectrolytes (synthetic polymers) are generally used as chemical conditioning agents. In addition to specific strength, other physical characteristics that significantly affect the sludge’s ability to be handled, compacted and dewatered are solids concentration, compressibility and particle size distribution [25].

**Table 1:** Chemical composition of WTS (Data from: Ahmed et al. [26], Abo-El-Enein et al. [27], Shamaki et al. [28], Altheman et al. [29], He et al. [30] and Liu et al. [31]).

Chemical Composition (%)	Countries					
	Iraq [26]	Egypt [27]	United Kingdom [28]	Brazil [29]	China [30]	Australia [31]
SiO_2_	36.29	36.51	10.28	42.00	43.75	26.43
Al_2_O_3_	27.92	22.21	44.24	35.00	36.57	28.27
Fe_2_O_3_	5.33	5.65	2.51	18.00	6.00	6.66
CaO	3.77	2.66	2.50	0.41	1.00	5.36
MgO	1.12	1.34	0.34	1.13	0.60	1.11
Na_2_O	1.31	1.35	0.15	0.04	-	-
K_2_O	1.81	0.49	0.43	0.95	2.00	1.23
SO_3_	0.55	0.08	1.24	0.86	2.04	0.48
P_2_O_5_	0.43	-	0.44	0.47	0.62	-

**Figure 1:**
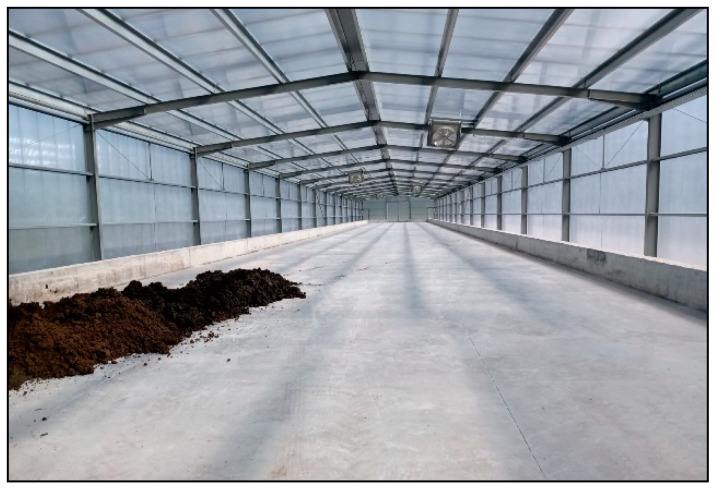
Solar drying greenhouse of the Areias de Vilar WTP.

WTS can be dewatered naturally, in drying beds or sludge lagoons, or using equipment to speed up the process, such as filter presses, vacuum filters, centrifuges and bags [25]. The most suitable method varies depending on several factors, such as area availability, climatic conditions, equipment costs and the operation and maintenance of the treatment system. In addition to these factors, one must also take into account the requirements defined by the control bodies in relation to the final concentration of solids. Generally, final disposal in landfills requires the sludge to be dewatered to a minimum solids content of 20% [32]. These values can be achieved using centrifuges or filter presses. After the mechanical dehydration process, in order to obtain a product with physical and chemical characteristics that increase its opportunities for valorization in the industry, many companies have started to use solar-powered greenhouse to carry out the final drying stage, ensuring greater efficiency and lower costs [33,34]. Figure 1 shows an image of the solar drying greenhouse installed at the Areias de Vilar WTP in Portugal when it received its first load of sludge from a centrifuge.

The geotechnical properties of the sludge must also be assessed to define alternative applications for this waste. The geotechnical analysis of alum sludge subjected only to thickening characterized the residue as a clay with high plasticity, high compressibility and very low permeability. The results were attributed to the large amount of water bound to the coagulant, the high affinity of the coagulant metal for water and the high organic content of the sludge [35], showing that untreated sludge is sometimes unsuitable for use in construction. However, by treating this sludge or incorporating it into other materials, the negative impact of these characteristics can be mitigated [36]. Other studies have also showed the similarity of WTS to clayey soil, based on the USCS (Unified Soil Classification System) [37,38,39]. However, despite this similarity, the concentration of organic matter and chemicals in WTS is higher than that in clay soils [40].

Due to its physicochemical characteristics, the use of WTS as a raw material for other processes is increasingly widespread. One promising application is its use as a supplementary cementitious material in civil construction, specifically to partially replace cement. This occurs because sludge contains high levels of SiO_2_ e Al_2_O_3_, giving them pozzolanic characteristics. Pozzolans are defined as a fine material, rich in silica or amorphous silica–alumina, capable of reacting with calcium oxide or hydroxide and forming compounds with cementing properties [41]. This property is enhanced in heat-treated WTS [42], where the crystalline content of silica and alumina is totally or partially broken down, forming a highly reactive transition phase.

Heat-activated alum sludge has been classified as a Class N pozzolan, based on its chemical composition. In addition to the chemical composition, the research also compared some physical properties of WTS, dried in an oven (105 °C for 24 h) and then calcined (800 °C for 2 h) with cement and another pozzolanic material [43] (Table 2). Heat treatment at temperatures between 600 °C and 800 °C has already been used successfully by other researchers to activate WTP sludge and make it viable for use as a pozzolan [42,44,45]. As the sludge is generally coarse-grained once it has been dried and calcined, it is necessary to crush and grind the waste to reduce its particle size and thus increase its pozzolanic potential [46].

A new treatment method applied to sludge consists of subjecting the waste to the rapid calcination process, in which the dried and ground sludge is calcined at high temperatures (800 to 900 °C) for a short period of time (0.5–1 s) and immediately cooled. The result is a more reactive material with potential for application in construction materials, obtained from a more sustainable method [47].

The sludge generated in WTPs is produced mainly in settling tanks, to a lesser extent in rapid filtration units and, in even smaller proportions, in preparation tanks for the chemical products used in the process.

The settling tanks accumulate the largest portion of solid waste, which represents 60 to 95% of the total sludge generated at the WTP, and the average volume of sludge generated daily in the decanting units of a full-cycle WTP can reach 3% of the volume of water treated by the plant [25]. The amount of sludge produced is directly related to the content of suspended solids presents in the raw water, removed during treatment process and the chemicals’ dosages applied in the unit processes can vary seasonally (rainy or dry-weather periods) or due to changes in raw water quality [2]. The types of treatment units where sludge are generated and the techniques used to remove this waste also affect the final quantity obtained.

Quantifying the production of sludge from a WTP is an essential step in planning sustainable alternatives for using this waste. This quantity can be estimated at the design stage through tests in pilot plants or laboratories, with the raw water to be treated [25] or, in WTPs in operation, by carrying out the system’s mass balance or using empirical formulas that relate parameters such as affluent flow rate, dosage (D) of coagulant and other products applied in the treatment, as well as the concentration of suspended solids in the raw water [48]. Katayama et al. [49] used the formula proposed by the American Water Association (AWWA) [50] to estimate the production of sludge in full-cycle WTPs that used aluminum sulphate or ferric chloride as a coagulant (Equation (1)) and compared the results with estimates made using the mass balance method. For the author, empirical formulas are widely used because of their practicality, but the mass balance method offers more precision and representativeness.

W = 86.4·Q·(D + SST + Dp + Dcap + 0.1Dcal)·10^−3^ (1)

where:

W—Dry solid production, (kg/day);

Q—Flow rate of water to be treated (L/s);

D—4.89·DAl or 2.9·DFe;

DAl—Aluminum sulphate dosage (mg/L);

DFe—Ferric chloride dosage (mg/L);

Dp—Polymer dosage (mg/L);

SST—Suspended solids in raw water (mg/L);

Dcap—Calcium hardness removed (mg/L CaCO_3_);

Dcal—Lime dosage (mg/L).

Precise information on the amount of WTS produced by each country is limited in the literature. However, there are records showing that in the USA, more than two million tons of dried sludge are produced annually [51]; in Italy, 750,000 tons of dewatered sludge are generated in one year, with an estimated transport cost of around 50 million euros/year [52]. The annual production of liquid sludge in Morocco has been estimated at one million tons, with the prospect of an increase of around 20% by 2030 [34]. There are reports that in Australia the annual generation of sludge from a WTP can reach 43,500 tons [20]. In India, the annual production of a WTP was estimated at 29,700 tons [2], while in Portugal, the WTPs managed by the Águas de Portugal Group (AdP) produced 18,076 tons of sludge in 2022, representing a per capita production of 26.2 kg/hab.year [53]. In this country, the cost associated with handling and transporting the sludge generated in a WTP reached 400,000 euros/year [54].

### Impacts of WTP Sludge on the Environment

The European Waste List (EWL) classifies WTS as non-hazardous solid waste [55]. In Brazil, these sludges have been classified as Class II A—non-inert waste [56], showing that if they are not properly treated and disposed, they can cause damage to the environment. This has captured the attention of researchers worldwide who are exploring sustainable management alternatives for this waste, particularly in supply systems in large urban centers, where the volume of sludge generated is significant and can lead to pollution problems if not properly disposed of. There is already published evidence in the literature regarding the toxicity of sludge and its negative effects on organisms in both soil and aquatic environments, predominantly due to the high concentrations of metals and organic compounds in WTS composition. Nevertheless, some scholars suggest that more in-depth studies should be carried out to clarify existing gaps and establish standards for use in specific areas [57,58].

In some countries, it is still common for WTS to be dumped directly into the environment. This is the case in Brazil, where most of the WTPs in operation discharge this waste, without proper treatment, directly into the water bodies located near the plants, directly affecting the quality of these water sources. Among the main impacts of this practice are an increase in the concentration of metals, mainly aluminum (Al) and iron (Fe) and in the concentration of suspended solids; alteration in the nutrient cycle, mainly phosphorus (P); development of anaerobic conditions in stationary or low-velocity waters; an increase in turbidity and color; a change in chemical composition; and siltation of receiving bodies, due to the increase in settleable solids and the possibility of groundwater contamination [59]. The accumulation of WTS on the benthic layer can inhibit the growth of some species of fish and aquatic organisms [60]. Studies conducted to assess the toxicity of alum sludge from 10 WTPs concluded that the water-soluble constituents present in the sludge, when discharged into receiving bodies, can affect the growth of algae [61]. Another study carried out with the aim of comparing the toxicity of iron sludge and alum sludge on *Daphnia similis* concluded that prolonged exposure to FeCl_3_ sludge caused mortality and decreased reproduction of these organisms, while alum sludge only caused reductions in reproduction [62]. Thus, WTS has the potential to cause negative effects on the soil, such as salinization, accumulation of metals, nitrate leaching, and on water, such as increased turbidity, consequent impairment of photosynthetic processes, increased organic matter, in addition to compromising aquatic flora and fauna [63].

Alum sludge contains high concentrations of Al [22]. The toxicity of this metal is still little known, but there is already research that shows some concerns about the actions of the element on aquatic organisms. Experiments with trout using different dosages of aluminum in different pH ranges led to the observation of physical changes in the fishes, such as generalized apathy and discouragement, a symptom of inability to keep their balance, changes in coloration and a decrease in perception [64]. The pH and organic matter content in the water influence the toxicity of this metal, which increases as the pH decreases [65]. Another study, which aimed to evaluate the oxidizing potential of aluminum sulphate in mouses, showed the role of aluminum in increasing the production and formation of free radicals and in the inflammatory action of the brain tissue of these animals [66]. Research carried out on individuals to explore the link between exposure to Al in drinking water and Alzheimer’s disease indicated that cognitive decline was more pronounced in individuals with a higher daily intake of Al, confirming that high Al consumption may be a risk factor for Alzheimer’s disease [67].

In any case, the environmental risks associated with WTP sludge are lower when compared to the sludge generated at wastewater treatment plants. This is because the raw water that is used as a source of supply needs to meet the requirements of public health bodies and must therefore be cleaner, in terms of the concentration of heavy metals, organic matter, levels of pathogens and other contaminants [17].

Another concerning factor is the large amount of sludge deposited in landfills, which can overload these units. Due to its high water content, which results in large volumes before being sent to their final destination, the sludge generated in WTPs needs to undergo dehydration treatment [59,68]. Generally, for final disposal in landfills, which is the most common destination in many parts of the world, it is required their dehydration reach a minimum solids content of 20% [32]. The aim is to reduce their high volume, to make them easier to handle and reduce transportation and storage costs [69]. In addition to the costs associated with this dehydration and storage stage, the transportation of WTS contributes to an increase in the material’s carbon footprint, due to the use of additional fuels [33].

## 3. Emerging Applications for WTS

**Figure 2:**
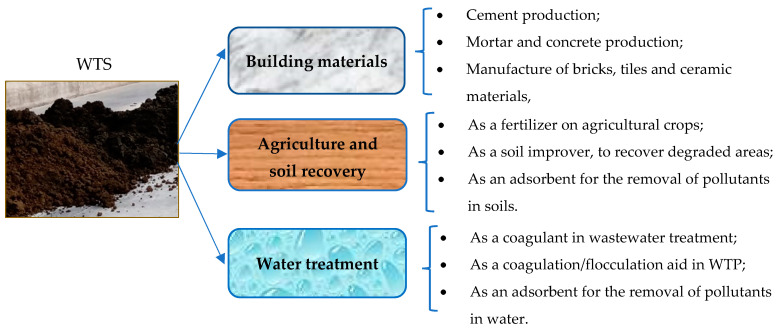
Emerging applications for WTP sludge.

Research points to various alternatives for the application of WTP sludge. The following have already been reported in the literature: its use as a soil improver [70]; as a waterproofing agent for landfill sites [71]; in agricultural crops [17]; for reuse as a coagulant in wastewater treatment [19] and as a coagulation/flocculation aid in WTP [72]; as an adsorbent for removing pollutants from soils and bodies of water [73,74,75]; for cement production, as an supplementary cementitious material [76,77]; and for the production of mortars [78], concrete [3,43,79], bricks, tiles and ceramic materials [80,81,82]. Recovering the aluminum present in the sludge for later reuse is also pointed out as a sustainable alternative in recovering this waste [83,84]. Figure 2 summarizes the main applications of WTS in different sectors.

Due to its composition, WTS has the potential to be applied in different areas. Alum sludge can be applied to the soil to improve its structure, porosity, water retention capacity, nutrient levels and organic compounds, because it contains significant amounts of organic matter and micro and macronutrients [70,85]. A lab-scale study using WTS to correct four types of soil at a rate of 1280 mg/ha proved that the treatment resulted in an increase in hydraulic conductivity and water retention, improving the soil’s physical properties [86].

Alum sludge has been used as an adsorbent to remove phosphorus from wastewater, and the removal capacities ranged from 2 to 43 mg P/g of sludge, depending on the experimental conditions and the characteristics of the sludge [23], and also showed promising results when used to remove emerging pollutants found in water. In this case, sludge used had a high concentration of activated carbon in its composition, which is normally used in treatment when it is desired to remove impurities from the water which affect taste and flavor. The results were promising for the removal of the steroid hormones 17β-estradiol and 17 β-ethinylestradiol [87].

Recovering aluminum metal for reuse, or even adapting the alum sludge for uses that require lower levels of aluminum, is a recovery option that offers both environmental and economic benefits. Research carried out to this end, using the alkaline process, managed to recover between 70% and 90% of the metal [83]. Another study used acid washing to remove aluminum from sludge and apply it to the soil for growing spinach and Japanese mustard. Acid washing (pH = 3) made it possible to reduce the aluminum content of the waste by up to 90%, which when mixed with the soil led to increased phosphate absorption by the plants and increased spinach size [65]. Acid washing, using a sulphuric acid solution with a molarity of 1.35 M, allowed 98% of the aluminum to be recovered from the sludge [84].

In the water treatment plant itself, the sludge can be used to assist in the coagulation/flocculation process, allowing savings to be made in the use of the coagulant applied in the WTP [72]. A study carried out in a laboratory setting used material recovered from alum sludge treated with sulphuric acid as a coagulant to treat water collected from a river. Most of the quality parameters of the treated water met the desired standards, indicating that the recovered product has the potential to be reused as a coagulant in a WTP [19].

Laboratory-scale experiments have also been reported where sludge (Fe or Al) has been used as an adsorbent to remove heavy metals from contaminated soils and bodies of water. The results obtained showed that small quantities of sludge were necessary for the adsorption of mercury (Hg) (19 mg/g) [73], of cadmium (Cd) (25 mg/g) [74] and of lead (Pb) (21.75 mg/g) [75].

The sludge has also been used as a coagulant in wastewater treatment plants (WWTPs) [88,89,90]. WTS applied in the post-treatment of upflow anaerobic sludge blanket (UASB) reactor effluents provided high removal efficiencies in terms of biochemical oxygen demand (BOD) (78%), chemical oxygen demand (COD) (74%) and suspended solids (SS) (84%), suggesting that this application is a promising option [91]. Another way of using WTS in wastewater treatment plants is to use it as a co-conditioning agent for dewatering wastewater biosolids [92,93].

### 3.1. Applications in the Construction Industry

#### 3.1.1. Cement Manufacturing

Among the main constituents of WTS are SiO_2_, Al_2_O_3_ and Fe_2_O_3_, which are also present in Portland cement, and this increases its potential for use as an SCM [9,94]. Supplementary cementitious materials are made up of siliceous, aluminosiliceous or calcium aluminosiliceous powders, used as partial substitutes for clinker in cement or as partial substitutes for Portland cement in concrete mixtures [15].

Iron mud mixed with lime powder in proportions of 1:3, 1:1 and 3:1 by weight and incinerated at 1000 °C for 4 h was used to produce cement. The properties of the cement produced were investigated, and the results confirmed that it could be used for masonry work in general, according to the American Society for Testing and Materials (ASTM). The best results were obtained using the mixture with a 1:1 ratio [95].

In China, alum sludge was used to replace clay in the production of clinker, and the effects of this addition on the sintering condition and cement quality were evaluated. Cement products made with this substitution met the Chinese National Standard for first-grade Portland cement [96]. Another study, also carried out in China, proposed replacing the siliceous raw material used in cement production with WTP sludge. The results showed that all samples with a content between 4% and 10% of sludge in their composition exhibited higher strength at 3 and 7 days, compared to the control samples [97].

More recently, other studies confirmed the potential for using WTS as an SCM for cement production. After being calcined, the sludge showed characteristics equivalent to normal pozzolanic material. The use of 14% and 35% slurry calcined at 600 °C met the compressive strength requirements to produce blended Portland cements, equivalent to CEM II/A-M, according to Standard EN 197-1 [76]. The chemical, physical, mineralogical and morphological characterization of the sludge was fundamental to verifying its potential for application as an SCM.

#### 3.1.2. Mortar and Concrete Production

Ruviaro et al. [77] showed it to be possible produce sustainable cement composites by replacing Portland cement with up to 20% WTP sludge, obtaining improved mechanical strength results compared to simple cement composites. The sludge used to prepare the cement paste was previously dried in an oven (105 °C for 24 h) and then calcined at 700 °C for 1 h. The research also confirmed that the CO_2-eq_ emissions associated with the production of 1 m³ of slurry with incorporated sludge decreased about 42% compared to the reference cement paste.

Hemkemeier et al. [78] used WTP sludge as fine aggregate to produce repair mortar. WTS previously dried and crushed was used to replace the fine sand in the mixture, in a proportion of 3% by mass, resulting in a mixture capable of providing more protection for steel reinforcement in aggressive environments with CO_2_ and Cl^-^, reducing the corrosion rate by around 70%.

WTS was tested as a pozzolanic material to partially replace cement in concrete. Sludge calcined at different temperatures (400, 500, 600 and 700 °C) was used, with a calcination time of 1 and 2 h. The study concluded that it is feasible to incorporate up to 30% mud by weight, and the most efficient calcination temperature was 600 °C for 1 h. The substitution resulted in an increase in compressive strength between 3% to 30%, when compared to the reference concrete (100% Portland cement), at 7 and 28 days [42]. Under these conditions, it was possible to reduce cement consumption in concrete by up to 200 kg/m^3^.

A mixture of calcined clay and ground limestone has already been used to partially replace cement, with promising results. Given that the chemical characteristics of WTS are like those of clay, another study sought to assess the pozzolanic activity of WTS and its potential as a substitute for calcined clay in concrete production, comparing results from ternary mixtures (heat-treated WTS, ground limestone and cement) and binary mixtures (heat-treated WTS and cement) with a reference test that used only cement. Ternary mixtures showed a synergistic effect, resulting in higher compressive strength compared to binary mixtures and single cement. A ternary mixture made up of 15% WTS and 7.5% ground limestone reduced Portland cement consumption by 34.7 to 38.4% while achieving compressive strength levels of between 45 and 60 MPa, indicating the potential for using this mixture to produce concrete [9]. The most promising results were obtained using sludge calcined at 700 °C for 1 h and ground for 1 h. The use of mineral additions to partially replace cement reduces the environmental impact of the construction industry, and WTP sludge is an attractive and sustainable alternative due to its geographical availability.

Alum sludge has also been tested to partially replace fine aggregates in concrete production. The research was carried out using mud dried at 105 °C for 24 h and mud heated to 300 °C for 3 h as a substitute for fine sand. The incorporation of up to 10% sludge, regardless of the heat treatment applied, provided higher mechanical strength and durability to the concrete [98]. Thus, the results suggest that it is environmentally sustainable to use kiln-dried sludge (105 °C), due to lower energy consumption. However, it was observed that sludge calcined at 300 °C achieved a better performance in all evaluated properties. Another study evaluated the influence of the use of WTS as a partial substitute for fine aggregates on the properties of conventional concrete in the hardened state, with a view to using it in the manufacture of interlocking paving blocks. The research used wet sludge, without any heat treatment, with the substitution in volume of the fine aggregate by WTS in the proportions of 5 and 10%. The addition of up to 5% sludge proved to be viable, as it did not cause any significant differences in axial strength or in the tensile strength tests compared to the reference sample. Thus, an attractive option for the use of WTS is its incorporation as a substitute for fine aggregate in concrete pieces with no structural purpose, such as interlocking blocks for paving and lightweight concrete for filling, concrete pipes and urban furniture, among other applications [79].

Several studies have shown that WTS activated by heat treatment and ground has been successfully used as an SCM, improving the properties of mortar and concrete and reducing the environmental impact of these materials [20,29,52,94,99]. Thus, the use of WTS in the production of supplementary cementitious material is considered a viable and sustainable alternative for civil construction [30,77]. However, to be used efficiently as an SCM, the physical and chemical properties of the waste must be evaluated, bearing in mind that these properties can directly affect the pozzolanic activity of the material, reflecting on the performance of the construction materials. Table 3 presents a summary of the main analyses and methods that are most used to characterize supplementary cementitious materials, with observations highlighted by researchers with regard to methodology [11,15,100,101].

#### 3.1.3. Manufacture of Bricks and Ceramic Products

Clay and WTP sludge have a similar chemical compositions, with significant levels of hydroxides and oxides of silicon, aluminum and iron. For this reason, WTS has been widely used to partially replace clay in the production of bricks and other ceramic products [80,82,102,103].

The alum sludge generated at the largest WTP in the city of Barcelona was evaluated as a composite for the manufacture of ceramic coating material. The sludge was dried by a spray-drying process, and the atomized waste powder was mixed with the clay in different percentages. By atomizing the sludge, it was possible to obtain a powder with a low organic content and a high calcium oxide content, which could be used in the production of ceramic tiles [80]. The tests were carried out on a laboratory scale but based on the results achieved and considering the associated environmental advantages, the WTP implemented the atomization drying process on a full scale and currently produces 30 tons of dry sludge per day. Spray-dried sludge has also been used in the cement industry to produce clinker, replacing clay [104].

Another study assessed the feasibility of using WTS in the production of structural ceramics. The research analyzed the influence of the dehydration method applied to the sludge and the proportion of waste used on the properties of the ceramic bricks produced. After being dehydrated, the sludge was dried and used to replace the clay in proportions of 5% to 20% by weight. The addition of 20% alum sludge, dehydrated using the freeze–thaw method, reduced the clay’s sensitivity to drying, reduced the ceramic’s density by 20% and also increased its compressive strength from 7.0 to 10.2 MPa [105]. The results confirmed that the sludge could be used to produce ceramic bricks, obtaining a product with improved properties.

The use of ferric sludge in ceramic products provided more promising results than that of alum sludge in terms of mechanical properties and the firing temperature of the bricks [82]. In addition, ferric sludge also acts as a natural pigment due to its high Fe_2_O_3_ content, giving bricks a reddish color [81].

#### 3.1.4. Geotechnical Works

Mixtures containing WTS and clayey and sandy soils were tested for application as barriers in bottom waterproofing layers, daily cover and final cover of landfills. The iron sludge was dehydrated in natural drying beds and mixed with the soils in proportions of 1:0.5 and 1:1 for the clay soil and 1:0.25 for the sandy soil. The compaction and permeability tests confirmed that all the mixtures were classified as low-permeability materials (with a permeability coefficient between 10^−10^ and 10^−9^ m/s) [106]. Therefore, they are suitable for use in landfills.

Another more recent study also evaluated the use of WTS as a water barrier and cover material for landfills and concluded that sludge can be used effectively for this purpose, as it provides similar results to the materials commonly used in engineering [71]. In Italy, a biosoil produced with WTP sludge and stabilized organic fraction from municipal solid waste showed good results for application as daily cover and final cover in landfills, in terms of chemical and physical properties, namely leaching tests [107].

Table 4 summarizes the main information about the works highlighted in this section, following the chronological order of the studies published.

## 4. Conclusions

In recent years, research in waste recovery has been very prominent in the scientific community, motivated mainly by global concern about the increasing demand for natural resources, the high amount of waste generated daily on the planet and the negative impacts it has on society from an environmental, social and economic point of view. In this context, since 2015, with the presentation by the UN of the 2030 Agenda for Sustainable Development, many countries around the world have implemented policies that encourage the circular economy, the aim of which is to reduce the consumption of natural resources through the reuse and recycling of materials, combined with the reduction of greenhouse gas emissions, improved energy efficiency and the use of cleaner technologies.

In this regard, research into more sustainable alternatives for the treatment and use of sludge generated at water treatment plants has also intensified. Over the last 15 years, the scientific community has proposed applications for WTS in various areas, based on evidence that confirms its feasibility. However, scholars agree on the need for a prior assessment of the waste’s physical and chemical properties to define the most efficient use.

## 5. Perspectives

In the field of construction, the use of WTS as a supplementary cementitious material to produce or partially replace cement is very promising, given the scarcity of industry waste currently used for this purpose and the geographical availability of sludge around the world.

However, despite the advances in research in this area, there are still issues that require further investigation. Thus, as part of future studies, we recommend approaches in the following aspects:

- Analysis of long-term durability indicators for applications such as SCMs in construction materials.
- Development of economic analyses, bearing in mind that the need for thermal treatment of sludge can increase costs.
- Development of life cycle analyses of materials with incorporated sludge, for better environmental assessment.
- Development of studies on the potential for valuing WTS for CO_2_ capture
- Development of full-scale studies.

## Figures and Tables

**Table 2:** Chemical composition and physical properties of cement, WTS and blast furnace slag (data from: Owaid et al. [43]).

Characteristics		Cement	Dry Sludge (105 °C for 24 h)	Calcined Sludge (800 °C for 2 h)	Blast Furnace Slag
Chemical composition (%)	SiO_2_	20.18	42.38	47.00	32.6
	Al_2_O_3_	5.23	35.03	41.94	12.57
	Fe_2_O_3_	3.34	4.94	4.86	0.24
	CaO	64.40	0.13	0.41	41.0
	MgO	1.80	0.29	0.40	6.04
	Na_2_O	0.07	0.10	0.09	0.39
	K_2_O	0.44	1.87	0.99	0.35
	SO_3_	2.98	0.14	0.10	1.31
	P_2_O_5_	-	0.26	0.28	-
	Loss on ignition	2.17	11.4	2.64	1.48
Physical properties	Specific gravity	3.12	2.34	2.53	2.83
	Specific surface (m^2^/kg)	338	1110	1160	739
	Average particle size (µm) (d_50_)	16.9	11.0	10.1	16.8
	Resistance activity index 7 days (%)	100	78	84	84
	Resistance activity index 28 days (%)	100	86	93	101.4

**Table 3:** Analyses and methodologies most used to characterize SCM.

Characteristics	Methodology	Observations
Chemical composition	X-ray Fluorescence Spectrometry (XRF)	Oxide composition—used in conjunction with phase composition to assess pozzolanic activity. Sample preparation can lead to the loss of volatile elements.
	Nuclear Magnetic Resonance Spectroscopy (NMR)	Atomic structure information (specific elements), applicable to amorphous phases.
Loss on Ignition—Volatiles	Thermogravimetric analysis (TGA)	Interference from reactions that cause weight changes (oxidation, decarbonation).
Mineralogy/ Phase Composition	X-Ray Diffraction (XRD)	Does not allow for differentiation between different amorphous phases. Amorphous phases provide diffuse signal, complex analysis.
Particle Size Distribution	Laser Diffractometry (LD)	Size ranges from 0.1 to 1000 µm. Sensitive to sample preparation; deagglomeration and dispersion can occur.
	Scanning Electron Microscopy (SEM)	Information about size, shape, texture and distribution. Requires more time and experience for sample preparation and data interpretation.
Specific Surface Area	N_2_ sorption—BET (Brunauer, Emmett and Teller)	More reliable than Blaine’s method.
	Air Permeability—Blaine	Standardized method for Portland cement. Not suitable for very fine or coarse powders.
Density	Helium (He) Gas Pycnometry	The sample must be dry, avoiding the measurement of apparent density.

**Table 4:** Summary of WTS applications in several building materials studies.

Study	Country	Application	Heat Treatment	Main Results
Chen et al. [97]	China, 2010.	Cement	Dry in an oven at 105 °C until constant weight.	Partially replacing the clay with WTS resulted in an “ecocement” with a higher compressive strength at 3 and 7 days than ordinary cement. By replacing the clay with 10% LETA, the strengths at 3 and 7 days were 13.0% and 5.6% higher, respectively.
Rodríguez et al. [104]	Spain, 2011.	Cement	Spray-drying	Atomized sludge was used as a raw material in the manufacture of clinker, replacing clay. The sludge showed high reactivity in the mixture. The clinker obtained had high proportions of allite (>70%), and its microstructure was like the reference clinker in terms of the size and composition of the allite and belite crystals.
Teixeira et al. [82]	Brazil, 2011.	Ceramic product	Oven-drying at 110 °C.	The use of WTS mixed with clay as a raw material resulted in bricks with characteristics within the standards defined for ceramics by Brazilian standards: flexural strength (bricks > 2.0 MPa, perforated bricks > 5.5 MPa), water absorption (perforated bricks < 25%), linear firing shrinkage (bricks < 6%) and apparent specific mass (>1.6 g/cm^3^).
Caniani et al. [107]	Italy, 2013.	Geotechnical work	-	Biosoil obtained from mixing WTS with the organic fraction of municipal solid waste proved to be suitable for use as daily cover and final cover in landfills. The mixture does not pose significant environmental risks, even at doses above 2000 tons ss/ha in single applications.
Kizinievič et al. [81]	Lithuania, 2013.	Ceramic product	Oven drying at 105 °C.	Incorporating 5% iron mud into the clay mixture, with the samples fired at 1000 °C or 1050 °C, resulted in an increase in the density and compressive strength of the ceramic body and in the reduction of water impregnation and effective porosity of the material produced. The incorporation of iron sludge resulted in a more intense tinting of the ceramic body, even in small proportions (5%).
Owaid et al. [43]	Malaysia, 2014.	Concrete	Drying in an oven at 105 °C for 24 h, followed by calcination at 800 °C for 2 h.	Binary mixtures containing 5%, 10% and 15% WTS as a partial cement substitute resulted in an increase in the concrete’s compressive strength at all ages compared to the control concrete: 3.4%, 8.4% and 9.3% at 7 days; 3.6%, 10% and 14.2% at 28 days; 5.4%, 9.3% and 12.5% at 56 days; and 3.1%, 6.3% and 9.8% at 90 days.
Benlalla et al. [102]	Morocco, 2015	Ceramic product	Sun-dried for 72 h, followed by oven-dried at 105 °C for 48 h.	The use of the WTS–clay mixture resulted in bricks with properties within the standards defined for ceramic bricks. All the bricks produced met the criteria regarding the degree of shrinkage during firing. With the incorporation of 5 to 10% by weight of WTS, the bricks fell into the first-class category in terms of water absorption and compressive strength standards.
Gastaldini et al. [42]	Brazil, 2015.	Concrete	Drying in an oven at 110 °C for 24 h, followed by calcination at 600 °C for 1 h.	The use of WTS as a pozzolanic material to partially replace cement in concrete (up to 30% by mass) resulted in an increase in compressive strength of between 3% and 30% compared to the reference concrete, at both 7 and 28 days. With the replacement, cement consumption was reduced by between 37 and 200 kg/m^3^ of concrete.
Wolff et al. [103]	Brazil, 2015.	Ceramic product	Drying in an oven at 110 °C for 2 h.	WTS, waste from the recovery of chemical reagents (including lime sludge) and fine granite waste were mixed (8 different compositions) and used to make bricks, replacing clay. Some of these mixtures showed promising results, indicating that they could be used in the production of interior tiles or acoustic bricks.
Tafarel et al. [79]	Brazil, 2016.	Concrete	Wet sludge, without any heat treatment.	Replacing 5% of the fine aggregate with WTS resulted in concrete with satisfactory axial compressive strength conditions for non-structural use.
Gonçalves et al. [106]	Brazil, 2017.	Geotechnical work	Drying in drainage beds (layer of gravel No. 3, overlaid by geotextile blankets) for 30 days.	Mixing WTS with clay soil (proportions 1:0.5 and 1:1) and with sandy soil (proportion 1:0.25) resulted in materials with a coefficient of permeability between 10^−10^ and 10^−9^ m/s, suitable for use in landfill works.
Ramirez et al. [3]	Brazil, 2017.	Concrete	Wet sludge, without any heat treatment.	The study evaluated the effects of partially replacing sand with WTS on the mechanical properties and water absorption of concrete. Substitution of up to 5% proved suitable for non-structural concrete applications.
Cremades et al. [80]	Spain, 2018.	Ceramic product	Spray-drying: maximum hot air temperature 350 °C	Atomizing the sludge resulted in a powder with a low organic content and a high concentration of lime. The waste was used to partially replace clay in the manufacture of ceramic material and the product obtained passed the leaching test (NEN-7345 [108]) and the accelerated degassing tests (European Space Agency standards PSS-01-702 [109] and PSS-01-729 [110]).
Hagemann et al. [9]	Brazil, 2019.	Concrete	Drying in an oven at 110 °C for 24 h, followed by calcination at 700 °C for 1 h.	Mixture containing WTS, ground limestone and cement resulted in higher compressive strength than binary mixtures (ground limestone and cement) and simple cement. The ternary mixture made up of 15% WTS and 7.5% ground limestone reduced Portland cement consumption in concrete production by up to 38.4%.
Godoy et al. [76]	Brazil, 2020.	Cement	Drying in an oven at 110 °C for 24 h, followed by calcination at 600 °C for 1 h.	The use of 14% and 35% WTS resulted in SCM that met the compressive strength requirements to produce Portland cement mixtures equivalent to CEM II/A-M (25 MPa 32 MPa), according to EN 197-1 [111].
Orlov et al. [105]	Russia, 2020.	Ceramic product	Freezing (−16 ± 2 °C) and thawing (20 ± 3 °C), followed by oven-drying at 105 ± 2 °C until constant weight was reached.	WTS pre-treated by the freeze–thaw method was used as an additive to partially replace clay in the production of ceramic bricks. The addition of WTS reduced the clay’s sensitivity to drying, decreased the ceramic’s density by 20% and increased its compressive strength from 7.0 to 10.2 MPa.
He et al. [30]	China, 2021.	Mortar	Drying in an oven at 105 °C for 24 h, followed by calcination at 900 °C for 2 h.	Mortar produced with 10% WTS as a partial substitute for the cement content showed higher compressive strength at 90 days than the reference sample
Kaish et al. [98]	Malaysia, 2021.	Concrete	Drying in an oven at 105 °C for 24 h.	Replacing the fine aggregate with WTS (at a rate of 10%) improved the density, mechanical properties and durability of the concrete.
Ruviaro et al. [77]	Brazil, 2021.	Cement paste	Drying in an oven at 105 °C for 24 h, followed by calcination at 700 °C for 1 h.	Replacing cement with up to 20% WTS resulted in pastes with comparable fresh-state properties and better mechanical strength than the reference paste. The CO_2_-eq emissions associated with the production of 1 m³ of paste decreased progressively, with a reduction of 42% for the highest level of WTS incorporation (45%)
De Carvalho et al. [99]	Australia, 2022	Cement paste	Drying in an oven at 105 °C for 24 h, followed by pyrolysis at 700 °C for 2 h.	Mixtures containing 1%, 2% and 5% “biochar” (WTS bio-coal) in place of cement resulted in pastes with slightly higher compressive strength at 28 days compared to the reference material. With the use of 10% biochar, pastes with a compressive strength similar to the reference material were obtained.
Altheman et al. [29]	Brazil, 2023.	Mortar	Drying in an oven at 105 °C; followed by calcination at 725 °C for 3 h.	Mixtures of WTP sludge and cement and WTS, blast furnace slag and cement were tested for use as pozzolanic material. The WTS and cement mixture resulted in mortar with compressive strength close to the minimum standards required (ABNT NBR 5752 [112]), reaching 89.2% of that of the reference sample. The use of WTS mixed with blast furnace slag resulted in mortar with compressive strength within the required standards.
Hemkemeier et al. [78]	Brazil, 2023.	Mortar	Drying in an oven at 110 °C for 24 h.	WTS partially replaced the fine aggregate in the production of repair mortar (3% in mass), resulting in a material that performed similarly to the reference sample in terms of carbonation and chloride penetration tests. The probability of corrosion was delayed by 25% and the corrosion rate of the steel reinforcement was reduced by 70%.

## Data Availability

Not applicable.
